# Calcium Oxide Derived from Waste Shells of Mussel, Cockle, and Scallop as the Heterogeneous Catalyst for Biodiesel Production

**DOI:** 10.1155/2013/460923

**Published:** 2013-12-18

**Authors:** Achanai Buasri, Nattawut Chaiyut, Vorrada Loryuenyong, Phatsakon Worawanitchaphong, Sarinthip Trongyong

**Affiliations:** ^1^Department of Materials Science and Engineering, Faculty of Engineering and Industrial Technology, Silpakorn University, Nakhon Pathom 73000, Thailand; ^2^National Center of Excellence for Petroleum, Petrochemicals and Advanced Materials, Chulalongkorn University, Bangkok 10330, Thailand

## Abstract

The waste shell was utilized as a bioresource of calcium oxide (CaO) in catalyzing a transesterification to produce biodiesel (methyl ester). The economic and environmen-friendly catalysts were prepared by a calcination method at 700–1,000°C for 4 h. The heterogeneous catalysts were characterized by X-ray diffraction (XRD), X-ray fluorescence (XRF), scanning electron microscopy (SEM), and the Brunauer-Emmett-Teller (BET) method. The effects of reaction variables such as reaction time, reaction temperature, methanol/oil molar ratio, and catalyst loading on the yield of biodiesel were investigated. Reusability of waste shell catalyst was also examined. The results indicated that the CaO catalysts derived from waste shell showed good reusability and had high potential to be used as biodiesel production catalysts in transesterification of palm oil with methanol.

## 1. Introduction

Recently, alternative energies have been focused worldwide because of recent energy crisis. Biodiesel is one of the interesting alternative fuels which can be produced from renewable sources [1]. It can be easily synthesized through transesterification of oil or esterification of fats using basic or acidic catalysts with heating functions [2]. Conventional homogeneous catalysts are expected to be replaced by heterogeneous catalysts mainly in the near future because of environmental constraints and simplifications in the existing processes. Solid catalysts could be easily separated from the reaction mixture by filtration and then reused [3]. Heterogeneous base catalysts eliminate the need for the neutralization of homogeneous base catalysts with acids and the removal of water in the commercial production of biodiesel, thereby lowering its production cost [4]. Among the heterogeneous catalysts that are being used in transesterification, calcium oxide (CaO) has a promising place, and many reports have been published on CaO-catalyzed transesterification using laboratory grade. It is cheap, abundantly available in nature (as limestone), and some of the sources of this compound are renewable (waste material consisting of calcium carbonate, CaCO_3_) [5]. However, the utilization of waste materials as heterogeneous catalysts has been of recent interest in the search for a sustainable process [6].

The catalyst synthesized with the waste shells opens door for renewable catalyst and at the same time recycles the waste generated. Utilization of these waste materials not only reduces the catalyst cost but also promotes environmentally benign process. These shells may also find their utility in other base catalyzed important organic reactions which will add value to the waste generated [7]. Mussel, cockle, and scallop are found in several parts of Thailand. The production of mussel, cockle, and scallop is quite large and the processing of this food also produces significant amounts of shell waste. In this paper, we utilized waste mussel, cockle, and scallop shells as the source of CaO for transesterification of palm oil into biodiesel. The effects of reaction time, reaction temperature, methanol/oil molar ratio, catalyst loading, and reusability of catalyst were systematically investigated.

## 2. Experimental

### 2.1. Materials

Palm oil was purchased from Morakot Industries Public Company Limited, Thailand. The molecular weight and density of the oil were measured to be 851.06 g/mole and 0.868 g/cm^3^, respectively. The mussel, cockle, and scallop shells were collected as wastes from university cafeterias. The waste shells were rinsed with water to remove dust and impurities and were then dried in an oven. All chemicals were analytical-grade reagents (Merck, >99% purity) and were used as received.

### 2.2. Catalysts Preparation

**Figure 1:**
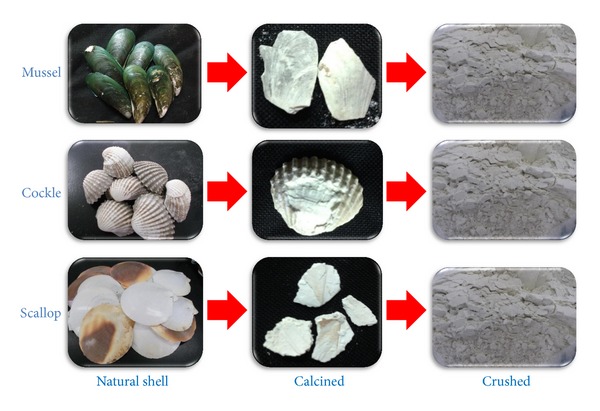
Preparation of CaO catalyst derived from waste shell (1,000°C).

The catalysts were prepared by a calcination method. The dried waste shells were calcined at 700–1,000°C in air atmosphere with a heating rate of 10°C/min for 4 h [2]. The solid result was crushed and sieved to pass 100–200 mesh screens. The products (38–75 *μ*m) were obtained as white powder. All calcined samples were kept in the close vessel to avoid the reaction with carbon dioxide (CO_2_) and humidity in air before being used.  Figure 1 illustrated the preparation process of waste shell-derived catalyst.

### 2.3. Catalysts Characterization

The X-ray diffraction (XRD) characterization of the waste shell-derived catalyst was performed on a Rigaku (MiniFlex II, England) based generator X-ray diffractometer using CuK*α* radiation over a 2*θ* range from 20° to 80° with a step size of 0.04° at a scanning speed of 3°/min.

The elemental chemical compositions of the materials were analyzed by X-ray fluorescence spectroscopy (XRF—Oxford, ED-2000, England) under energy dispersive mode for precise measurement of both light and heavy elements.

The microstructures of the calcined waste shells were observed by a scanning electron microscope (SEM). The SEM images of the representative sample were obtained from a Camscan-MX 2000 (England) equipped with an energy dispersive spectroscope (EDS).

To evaluate the surface area, mean pore diameter, and pore volume, adsorption-desorption of nitrogen (N_2_) at 77 K was carried out by a Quantachrome Instrument (Autosorb-1 Model No. ASIMP.VP4, USA). Before taking adsorption data, degassing at 120°C and a residual pressure of 300 *μ*m Hg for 24 h was performed using the degas port. The surface area was calculated using the Brunauer-Emmett-Teller (BET) equation and the mean pore diameter and pore volume was obtained by applying the Barret-Joyner-Halenda (BJH) method on the desorption branch [8].

### 2.4. Transesterification of Palm Oil

The synthesis of biodiesel from palm oil and methanol was carried out in a 500 mL glass reactor equipped with condenser and mechanical stirrer at atmospheric pressure. The effects of reaction time (2 to 6 h), reaction temperature (50 to 70°C), methanol/oil molar ratio (6 to 18), catalyst loading (5 to 25 wt.%), and reusability of catalyst (1 to 4 times) on the conversion to biodiesel were studied. After a certain period of time, a known amount of sample was taken out from the reactor for analysis. All experiments were repeated 3 times and the standard deviation was never higher than 7% for any point.

Composition of the fatty acid methyl ester (FAME) was analyzed with gas chromatograph-mass spectrometry (GC-MS QP2010 Plus, Shimadzu Corporation, Japan) equipped with a flame ionization detector (FID) and a capillary column 30 m × 0.32 mm × 0.25 *μ*m (DB-WAX, Carbowax 20 M). Yield of FAME was calculated by:

(1) $$\begin{array}{c} \text{Yield}\;\left(\%\right)=\frac{m_{i}A_{b}}{A_{i}m_{b}}\times 100, \end{array}$$

where *m*_*i*_ is the mass of internal standard added to the sample, *A*_*i*_ is the peak area of internal standard, *m*_*b*_ is the mass of the biodiesel sample, and *A*_*b*_ is the peak area of the biodiesel sample [9, 10]. The physical and chemical properties of FAME including kinematic viscosity, density, flash point, cloud point, pour point, acid value, and water content were analyzed according to ASTM methods [11].

## 3. Results and Discussions

### 3.1. Characterization of Waste Shell and CaO Catalyst

**Figure 2:**
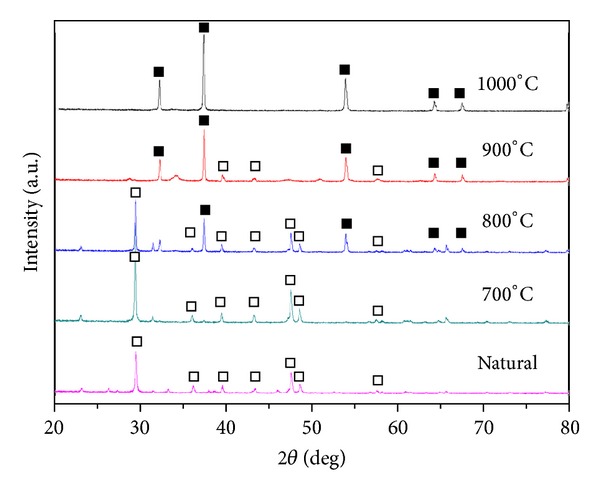
XRD patterns of natural and calcined mussel shell (□: CaCO_3_, ■: CaO).

**Figure 3:**
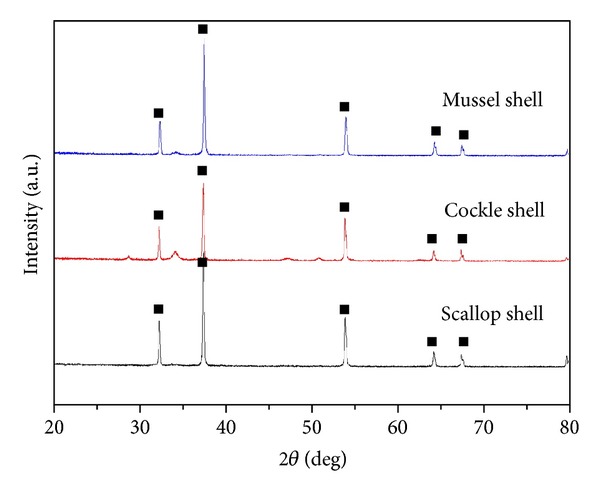
XRD patterns of waste mussel, cockle, and scallop shell calcined at 1,000°C (■: CaO).

The XRD patterns of natural and calcined mussel shell are given in Figure 2. XRD results revealed that the composition of natural mussel shell mainly consists of CaCO_3_ with the absence of CaO peak, as indicated by diffraction peak at 2*θ* around 29.2° [5]. However, with the increase in calcination temperature, CaCO_3_ completely transforms to CaO by evolving the carbon dioxide (CO_2_). The composition of calcined catalyst at and above 900°C mainly consists of the active ingredient (CaO). Narrow and high intense peaks of the calcined catalyst define the well-crystallized structure of the CaO catalyst [6]. The major component of the calcined waste shell at 1,000°C for 4 h was CaO species (Figure 3). The result reveals sharp XRD reflections with (1 1 1), (2 0 0), (2 2 0), (3 1 1), and (2 2 2) orientations, implying that the calcined material was well crystallized during the heat treatment process [2].

The chemical compositions of the catalyst are presented in Table 1. The major mineralogical component is CaO. The waste mussel, cockle, and scallop shells-derived catalysts have concentration of CaO 98.37, 99.17, and 97.53 wt.%, respectively.

**Figure 4:**
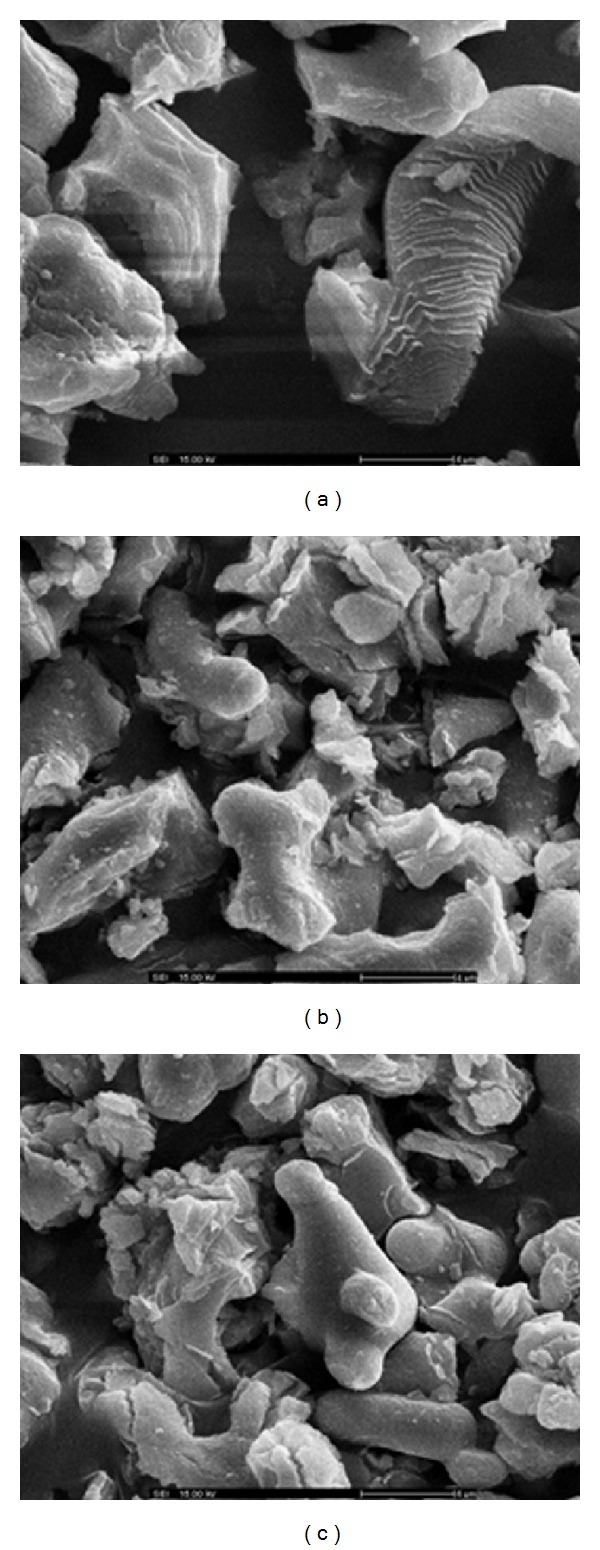
SEM images of (a) mussel shell, (b) cockle shell, and (c) scallop shell calcined at 1,000°C.

The morphology of waste mussel, cockle, and scallop shell calcined at 1,000°C was examined by SEM (Figure 4). The natural shell displays a typical layered architecture [12]. With the calcination temperature rising from 700 to 1,000°C, the microstructures of natural shell are changed significantly from layered architecture to porous structure [13]. The calcined cockle shell and scallop shell showed similar particle morphology with the calcined mussel shell. The calcined waste shells were irregular in shape, and some of them bonded together as aggregates. However, the smaller size of the grains and aggregates could provide higher specific surface areas. Since all samples are considered to be less-porous or even nonporous, the size of the particle should directly respond to the surface area [14].

The physical properties of the CaO catalyst are summarized in Table 2. The waste mussel shell-derived catalyst had a large surface area (89.91 m^2^/g) and pore volume (0.130 cm^3^/g), and presented a uniform pore size. The cockle and scallop shell-derived catalysts present lower values for surface area (59.87 and 74.96 m^2^/g, resp.) and pore volume (0.087 and 0.097 cm^3^/g, resp.) related to mussel shell. It can be seen that the heterogeneous catalyst resulted in a strong increase in the active sites [15]. This assumption is supported by the SEM images of catalyst.

### 3.2. Effect of Reaction Variables

The yield of biodiesel was affected by reaction variables, such as reaction time, reaction temperature, methanol/oil molar ratio, catalyst loading, and reusability of catalyst. The reaction variables were associated with the type of catalysts used [16]. Therefore, the effect of reaction variables was studied in the presence of waste shell-derived catalyst. For the following reactions, all the catalysts were prepared by calcinning waste shells at 1,000°C for 4 h.

**Figure 5:**
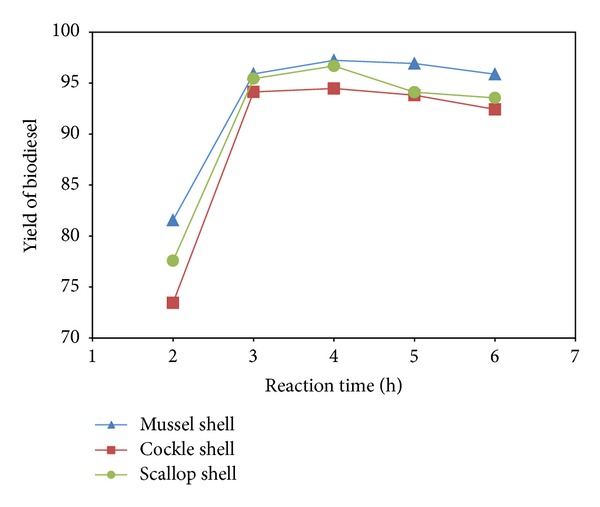
Effect of reaction time on % yield of biodiesel.

The effect of reaction time on the conversion of palm oil to biodiesel was investigated. Reaction time is one of the key parameters during the transesterification carried out in glass reactor.  Figure 5 shows an increase in the yield with time from 2 to 3 h with a catalyst amount of 10 wt.% relative to oil and a methanol/oil molar ratio of 9 : 1. The maximum yields of 97.23, 94.47, and 96.68% were obtained in 4 h at 65°C for mussel, cockle and scallop shell, respectively. In the initial stages of the transesterification reaction, production of biodiesel was rapid, and the rate diminished and finally reached equilibrium [17] in about 4 h. This can be explained by that transesterification reaction between oil and alcohol is reversible, when the reaction time is long enough [18].

**Figure 6:**
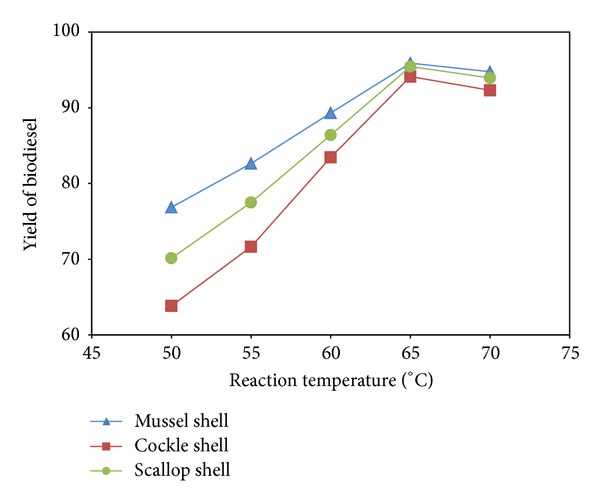
Effect of reaction temperature on % yield of biodiesel.

In general, the reaction temperature can influence the reaction rate and yield of biodiesel. The transesterification of triglyceride (TG) with methanol to methyl ester was carried out over the catalysts of CaO at reaction temperature 50–70°C. The % yields of biodiesel after 3 h of reaction time are shown as a function of temperature in Figure 6. The yields of biodiesel were obviously rising from 76.85 to 95.90% for mussel shell, 63.83 to 94.13% for cockle shell, and 70.14 to 95.44% for scallop shell with the increasing temperature from 50 to 65°C. The effect of reaction temperature on promoting transesterification can be explained due to endothermic reaction [18]. The highest yield rate was obtained at the reaction temperature of 65°C. When the reaction temperature continued to increase over 65°C, the yield of biodiesel was decreased. The reaction temperature consumedly exceeds the boiling point of methanol such as 70°C, and the methanol will quickly vaporize and form a large number of bubbles, which inhibits the reaction on the two-phase interface [19]. Moreover, in order to save energy, it is necessary to choose the relative low temperature. Therefore, the optimum reaction temperature for the transesterification of TG to methyl ester is considered to be around 65°C.

**Figure 7:**
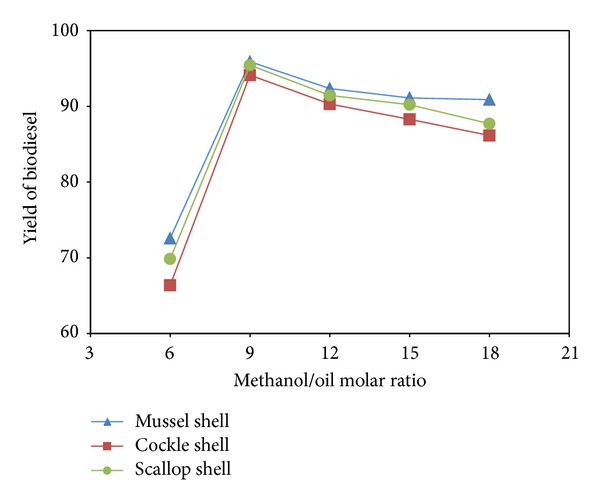
Effect of methanol/oil molar ratio on % yield of biodiesel.

The excess of methanol is necessary because it can increase the rate of methanolysis. Normally, stoichiometric molar ratio of methanol to TG is near 6 : 1 when the alkali-catalyzed process is used. However, it increases to 30 : 1, even 50 : 1, in the acid-catalyzed one to ensure high conversion [20]. The methyl ester content increased significantly when the methanol/oil molar ratio was changed from 6 to 18 (Figure 7). The high amount of methanol promoted the formation of methoxy species on the CaO surface, leading to a shift in the equilibrium in the forward direction, thus increasing the rate of conversion up to 95.90, 94.13, and 95.44% for mussel, cockle and scallop shell, respectively. However, further increases in the methanol/oil molar ratio, did not promote the reaction. It is understood that the glycerol would largely dissolve in excessive methanol and subsequently inhibit the reaction of methanol to the reactants and catalyst, thus interfering with the separation of glycerin, which in turn lowers the conversion by shifting the equilibrium in the reverse direction [21]. Therefore, the optimum molar ratio of methanol to oil was 9, which is more than the practical methanol to oil molar ratio for homogeneous transesterification [22].

**Figure 8:**
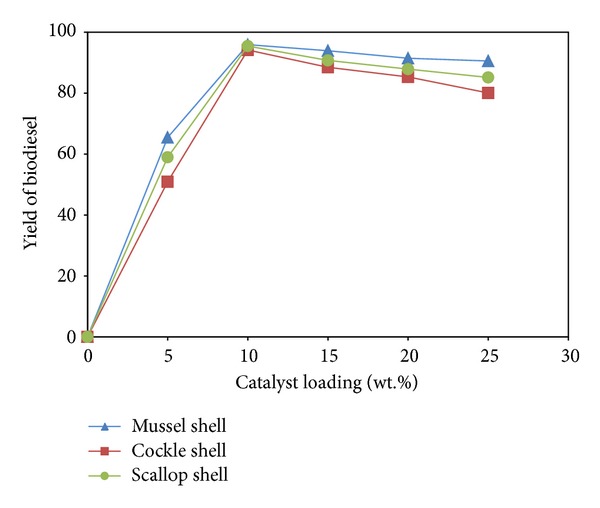
Effect of catalyst loading on % yield of biodiesel.

Figure 8 reveals the effect of catalyst loading on the methyl ester formation in the transesterification of palm oil over waste shell-derived catalyst. In the absence of catalyst, there was no methyl esters formed in the reaction. Applying the catalyst amount of 10 wt.%, the highest yields of 95.90, 94.13, and 95.44% were obtained within 3 h for mussel, cockle and scallop shell, respectively. Reducing the catalyst loading to 5 wt.% decreased the methyl ester content to ca. 50.92–65.45%. This result implies that the transesterification of TG is strongly dependent on the amount of basic sites [23]. The loadings of 15–25 wt.% created catalyst accumulation on the wall of the glass reactor, possibly contributing to diffusional problems during reaction and, therefore, lowering the activity [24]. From this study, we can conclude that the suitable amount of CaO required for the transesterification of palm oil is 10 wt.%.

**Figure 9:**
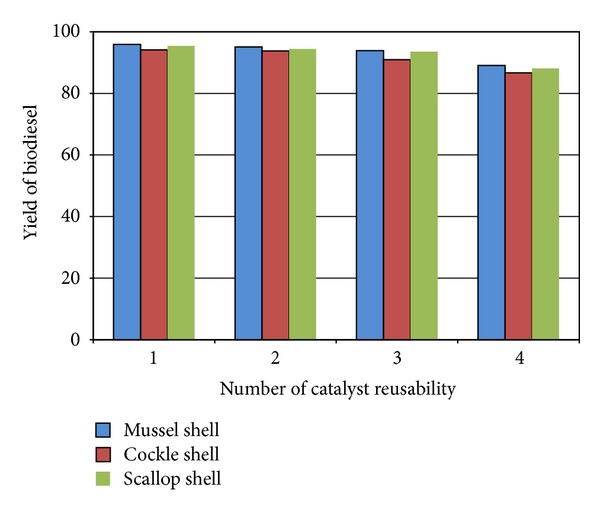
Effect of reusability of catalyst on % yield of biodiesel.

The reusability of catalyst is examined by carrying out reaction cycles. When transesterification reaction finished, the catalyst is separated from the mixture and used again without any subsequent treatment in a second reaction under the same conditions as before. It is found that the prepared catalyst is active for 3 reaction cycles, with yield above 90%. After 3 reaction cycles, the biodiesel yield lowers to 90% (Figure 9). Catalyst deterioration is probably due to the change of catalyst surface structure. Calcium oxide is transformed to calcium hydroxide gradually due to the moisture in the reactants, which deteriorate the activity of catalyst [25, 26]. However, the activity can be recovered after calcination in air at 600°C [13].

### 3.3. Fuel Properties of Methyl Ester

The fuel properties of methyl ester obtained in this work are summarized in Table 3. It can be seen that most of its properties are in the range of fuel properties as described in the latest standards for biodiesel [27].

## 4. Conclusions

Using cost-effective and environment-friendly catalysts is particularly useful for the production of biodiesel. The waste shells are used as the catalyst for this process. This catalyst contains CaCO_3_ which is converted to CaO after calcination at temperatures 1,000°C for 4 h. The optimum conditions, which yielded a conversion of palm oil of nearly 95% for all waste shell-derived catalysts, were reaction time 3 h, reaction temperature 65°C, methanol/oil molar ratio 9, and catalyst loading 10 wt.% with pressure 1 atm in glass reactor. The experimental results show that CaO catalyst had excellent activity and stability during transesterification. The catalyst was used for 4 cycles and apparent low activity loss was observed. The fuel properties of the biodiesel so obtained meet all biodiesel standards. As a solid catalyst, CaO can decrease the cost of biodiesel and the steps of purification. It has potential for industrial application in the transesterification of palm oil to methyl ester.

## Figures and Tables

**Table 1:** Chemical compositions of waste shell-derived catalyst.

Compound	Concentration (wt.%)		
	Mussel shell	Cockle shell	Scallop shell
CaO	98.367	99.170	97.529
Na_2_O	0.937	0.438	0.565
SO_3_	0.293	0.117	1.568
P_2_O_5_	0.163	0.096	0.204
SrO	0.158	0.132	0.107
ZrO_2_	0.046	—	0.027
Cl	0.037	—	—
Fe_2_O_3_	—	0.026	—

**Table 2:** The physical properties of waste shell-derived catalyst.

Physical property	Derived catalyst		
	Mussel shell	Cockle shell	Scallop shell
Surface area (m^2^/g)	89.91	59.87	74.96
Pore volume (cm^3^/g)	0.130	0.087	0.097
Mean pore diameter (Å)	34.55	25.53	30.55

**Table 3:** The fuel properties of biodiesel.

Fuel property	Derived catalyst		
	Mussel shell	Cockle shell	Scallop shell
Kinematic viscosity (mm^2^/s) at 40°C	4.4	4.6	4.5
Density (g/cm^3^) at 80°C	0.877	0.878	0.878
Flash point (°C)	164	165	164
Cloud point (°C)	11	12	11
Pour point (°C)	7	8	8
Acid value (mg KOH/g oil)	0.47	0.67	0.55
Water content (%)	0.02	0.03	0.02
